# Genetic Dissection of Mature Root Characteristics by Genome-Wide Association Studies in Rapeseed (*Brassica napus* L.)

**DOI:** 10.3390/plants10122569

**Published:** 2021-11-24

**Authors:** Sani Ibrahim, Keqi Li, Nazir Ahmad, Lieqiong Kuang, Salisu Bello Sadau, Ze Tian, Lintao Huang, Xinfa Wang, Xiaoling Dun, Hanzhong Wang

**Affiliations:** 1 Key Laboratory of Biology and Genetic Improvement of Oil Crops, Oil Crops Research Institute of the Chinese Academy of Agricultural Sciences, Ministry of Agriculture, Wuhan 430062, China; sibrahim.bot@buk.edu.ng (S.I.); likeqi1218@sina.com (K.L.); nazir_aup@yahoo.com (N.A.); kuanglieqiong@163.com (L.K.); tianze0825@163.com (Z.T.); hlt1406859197@outlook.com (L.H.); wangxinfa@caas.cn (X.W.); wanghz@oilcrops.cn (H.W.); 2 Department of Plant Biology, Faculty of Life Sciences, College of Physical and Pharmaceutical Sciences, Bayero University, Kano, P.M.B. 3011, Kano 700006, Nigeria; 3 State Key Laboratory of Cotton Biology, Institute of Cotton Research, Chinese Academy of Agricultural Sciences, Anyang 455000, China; sbsadau.ste@buk.edu.ng

**Keywords:** *Brassica napus*, root traits, GWAS, QTL, candidate genes

## Abstract

Roots are complicated quantitative characteristics that play an essential role in absorbing water and nutrients. To uncover the genetic variations for root-related traits in rapeseed, twelve mature root traits of a *Brassica napus* association panel were investigated in the field within three environments. All traits showed significant phenotypic variation among genotypes, with heritabilities ranging from 55.18% to 79.68%. Genome-wide association studies (GWAS) using 20,131 SNPs discovered 172 marker-trait associations, including 103 significant SNPs (−log10 (*p*) > 4.30) that explained 5.24–20.31% of the phenotypic variance. With the linkage disequilibrium r^2^ > 0.2, these significant associations were binned into 40 quantitative trait loci (QTL) clusters. Among them, 14 important QTL clusters were discovered in two environments and/or with phenotypic contributions greater than 10%. By analyzing the genomic regions within 100 kb upstream and downstream of the peak SNPs within the 14 loci, 334 annotated genes were found. Among these, 32 genes were potentially associated with root development according to their expression analysis. Furthermore, the protein interaction network using the 334 annotated genes gave nine genes involved in a substantial number of interactions, including a key gene associated with root development, *BnaC09g36350D.* This research provides the groundwork for deciphering *B. napus*’ genetic variations and improving its root system architecture.

## 1. Introduction

Root system architecture (RSA), which is made up of structural components such as root number, length, spread, and length of lateral roots, among others, shows a lot of flexibility in response to environmental changes [1]. RSA is crucial for plant anchorage and efficient absorption of water and nutrients and can significantly affect fertilizer use and yield in crops worldwide. Because of the diverse nature of the surrounding world, RSA is highly plastic and can be made up of various root types, each with its own set of functions [2]. RSA reacts to external environmental factors such as nutrients, soil moisture, pH, temperature, and microbial communities under poorly understood genetic regulation [3]. Comprehending RSA and the processes that lead to it will enable researchers to manipulate and exploit different root traits to help plants adapt to changing climates and increase yields [4].

The genome-wide association study (GWAS) method is commonly utilized to determine associations between molecular markers or candidate genes and investigated traits in a natural population based on linkage disequilibrium (LD) [5]. It has many advantages over conventional linkage mapping, including more precise positioning and visualization, simultaneous evaluation of multiple alleles at a locus, and minimizing the need for linkage group formation [6]. It has been used to investigate complex agronomic traits in crops such as rice, soybean, maize, sesame, rapeseed, barley, wheat, and others [7,8,9,10,11,12,13,14,15,16,17]. Nevertheless, quantitative studies on crop roots are limited compared to other important aboveground agronomic traits due to the intricacy of root architecture [18,19]. Moreover, since it typically requires a mixture of field, laboratory-based screens, and glasshouse experiments, measuring root traits in a crop breeding program is tedious, time-consuming, and costly [20]. The primary limitations are the difficulties associated with examining complete roots and phenotyping root traits of many genotypes in similar conditions [21]. To conquer these limitations, in the last decade, a variety of comprehensive image analysis systems have been developed to enhance the throughput and precision of RSA trait measurements [21,22].

Rapeseed (*Brassica napus* L.; Brassicaceae) is the third-largest oilseed crop after soybeans and palm. According to the different requests for the temperature at the vernalization stage, *B. napus* is categorized into three types; winter, semi-winter, and spring [23]. Understanding the effect of rapeseed root development is of great significance to increase the yield of rape. However, only a few studies in *B. napus* have converged on root traits [8,21,24,25], especially the critical function of root morphology in phosphorus, boron, and nitrogen uptake ability [26,27,28]. For example, Shi et al. [27] discovered 38 quantitative trait loci (QTL) linked to root architectural traits and biomass under high and low phosphorous (P) levels in *B. napus*. Additionally, a linkage mapping analysis was conducted by Duan et al. [29] to identify nine quantitative trait loci (QTLs) for the root angle at LP. Furthermore, 28 stage-specific and 23 persistent QTL related to root growth, explaining the genetic variance of 5.1 to 36.2%, were detected by dynamic unconditional and conditional QTL mapping in rapeseed [21]. On chromosome A01 (24.7 Mb), a QTL mapping for root vigor was discovered, explaining 16.3% of the phenotypic variance [24]. In addition, several genes controlling root development were co-localized with genomic regions associated with root-related traits or nutrient utilization efficiency in rapeseed. Examples included genes controlling root and root-hair growth localized with proximity to chromosomal areas related to leaf P content [30], and 17 genes linked to roots in the genomic regions of *QTL.A10* [31]. According to Tong et al. [32], the *BnNRT2.1* generated is primarily expressed in roots and was up-regulated during N shortage stress.

*B. napus* root architecture differs considerably depending on growth habits; the root system of winter canola is more robust and compact than the root system of spring rapeseed [23]. In the present study*,* a diverse association population consisting of 338 *B. napus* accessions were genotyped with a new *Brassica napus* 50K Illumina Infinium SNP array [33], including spring, winter, and semi-winter accessions. In three different environments, WH17, WH19, and WH20, root-related traits of the association population were studied. The objective of this study was to exploit the GWAS approach to reveal the genetic basis of roots-related traits and find out the possible genes controlling the RSA in rapeseed.

## 2. Results

### 2.1. Phenotypic Variations in Root Traits

A total of twelve root-related traits, primary root length (PRL), the diameter of root (DMR), root fresh weight (RFW), total root length (TRL), root dry weight (RDW), total root surface area (TSA), total root volume (TRV), total root number (TRN), total root length above 0.5 cm (TRL0.5), total root surface area above 0.5 cm (TSA0.5), total root volume above 0.5 cm (TRV0.5), and total root number above 0.5 cm (TRN0.5) were measured (Table 1). The frequency distributions of all the investigated traits showed a normal distribution or approximate normal distribution, with a right skew in the entire three environments, except for PRL showing a left skew (Figure S1). For all the traits, a considerable phenotypic variation was observed among the genotypes, with the coefficient of variation (CV) values ranging from 9.36% to 67.50% (Table 2). PRL with each CV of 15.44%, 10.33%, and 9.36% in WH17, WH19, and WH20, respectively, was the minor variation of all the results; TRN with each CV of 44.99%, 48.32%, and 67.50% was the most considerable variation of all the results. Furthermore, broad-sense heritability (H^2^) was calculated across the three environments. The results showed that all the traits displayed a moderate to high heritability, ranging from 55.18% to 79.68% for TRV0.5 and TRL, respectively (Table 2). This suggested that the phenotypic variations of root phenotypes were mainly derived from genetic factors, and therefore, they are apt for further GWAS analysis. However, The performance of individual genotypes over the course of three years was also investigated using phenotypic variations of the 12 traits (Table S5).

### 2.2. Correlation Analyses between Root Traits

**Figure 1:**
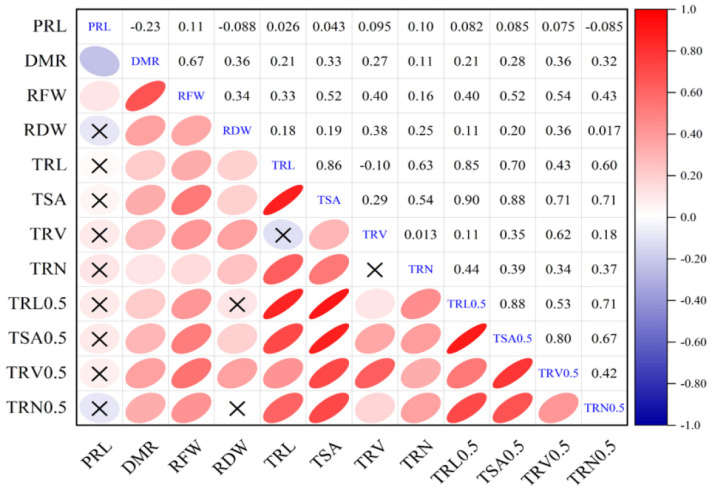
Correlation analyses between root-related traits. The plots on the diagonal line show the traits. Above the diagonal line are Pearson correlation coefficient values between traits. The plots below the diagonal line indicate the strength of the correlation coefficient values; ellipses with “X” inside them depict insignificant correlation. Significant differences at *p* < 0.05. Refer to Table 1 for the definition of terms.

Correlation coefficients between root traits were evaluated to reveal their contact (Figure 1). DMR and RFW were positively correlated with all the eight image root morphological traits (r = 0.11 to 0.36, 0.16 to 0.54; *p* < 0.01). Furthermore, DMR and RFW displayed higher correlations with root morphological traits with a diameter above 0.5 cm than that with all roots, except for the correlation between DMR and TSA0.5. For the image root morphological traits with all roots, positive significant correlation coefficients were observed between TRL and TSA, TRL and TRN, TSA and TRV, and TSA and TRN (r = 0.86, 0.63, 0.29, and 0.54 respectively; *p* < 0.01). Similarly, a high significant positive correlation was observed in rice [34]. However, no or low correlations were observed between TRL and TRV and TRV and TRN (r = −0.10, 0.02). For another classification of image root morphological traits with root diameter above 0.5 cm, TRL0.5, TSA0.5, TRV0.5, and TRN0.5 displayed higher positive significant correlations with each other, ranging from 0.41 to 0.88 (*p* < 0.01). The results may be due to phenotypic survey errors for lateral roots with a diameter less than 0.5 cm. DMR and PRL were positive significantly correlated with RFW (r = 0.67, 0.19 respectively; *p* < 0.01). Interestingly, PRL displayed a low but negative significant correlation coefficient with DMR (r = −0.25, *p* < 0.01), with no or low correlations with other root-related traits. The results showed stability for image root morphological traits with a diameter above 0.5 cm, and also revealed genetic relative among the investigated root traits. Table S9, on the other hand, showed the correlations between the analyzed root-related traits of the individual environments.

### 2.3. Analyses of Linkage Disequilibrium (LD), Population Structure, and Relative Kinship of the Association Panel

**Figure 2:**
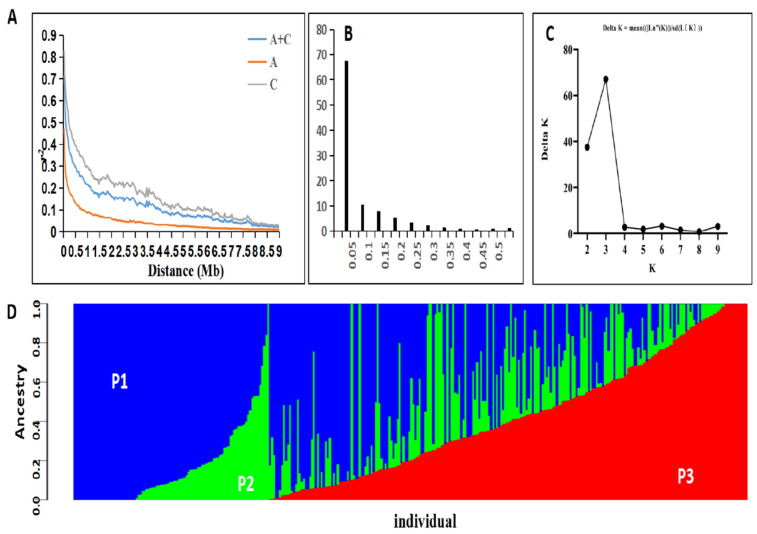
Linkage disequilibrium (LD) analysis and population structure and relative kinships of the 338 *B. napus* accession populations. (**A**) Linkage disequilibrium decay calculated by squared correlations of allele frequencies (r^2^) against the distance between polymorphic sites in the A subgenome (red), C subgenome (grey), and A + C subgenome (blue). (**B**) Distribution of pairwise relative kinship estimates in the entire population. (**C**) The log-likelihood of the data (LnP[D]) of possible clusters (K) from one to 10. (**D**) Structure of the population based on K = 3. Subpopulations 1, 2, and 3 are represented by P1, P2, and P3, respectively.

The *Brassica* 50K Illumina Infinium SNP array contained 45,708 SNPs. Among these, the probe sequences of 28,142 SNPs were uniquely matched in the *Brassica_napus*_v4.1 Damor genome with an e-value threshold of e^−10^. The distribution of the SNP markers was not evenly across the entire genome. In the association population used, A03 (1103 SNPs) and C03 (2026 SNPs) had the highest number of SNPs in the A-sub and C-sub genomes, respectively, whereas A08 (599 SNPs) and C05 (911 SNPs) had the smallest number of SNPs (Table S6). This is in line with Li et al. [35], who found that the C genome (C04) had the highest marker density (2104 SNPs), and Wu et al. [36] also found that the C genome (C04) had the highest marker density (2104 SNPs) (1004 SNPs). Additionally, A02 and C09 (35.3 kb/SNP and 51.1 kb/SNP) had the highest marker density in the A and C genomes, respectively (Table S6). Moreover, between all the pairs of SNP markers, the linkage disequilibrium (LD) was estimated as r^2^, the squared Pearson correlation coefficient. When the linkage disequilibrium (LD) decays to half, the linkage disequilibrium (LD) decay of A sub-genome was about 0.10–0.15 Mb, while that of the C sub-genome was about 0.45–0.50 Mb (Table S6). Consistent with previous reports [8,36,37], the C subgenomes linkage disequilibrium (LD) value was significantly larger than that of the A sub-genome in the natural population (Figure 2A). Approximately 67.3% of the kinship coefficients between individual accessions were equal to zero, and 95.3% were less than 0.2, suggesting a weak kinship for most accessions in the natural population (Figure 2B). Based on the population structure analysis, the 338 accessions could be classified into three main sub-populations: P1, P2, and P3 (Figure 2C,D). The P1 subpopulation included 190 accessions belonging to the semi-winter *B. napus* type. There were 37 winter accessions in the P2 subpopulation and 111 spring accessions in the P3 subpopulation.

### 2.4. Marker-Trait Association

**Figure 3:**
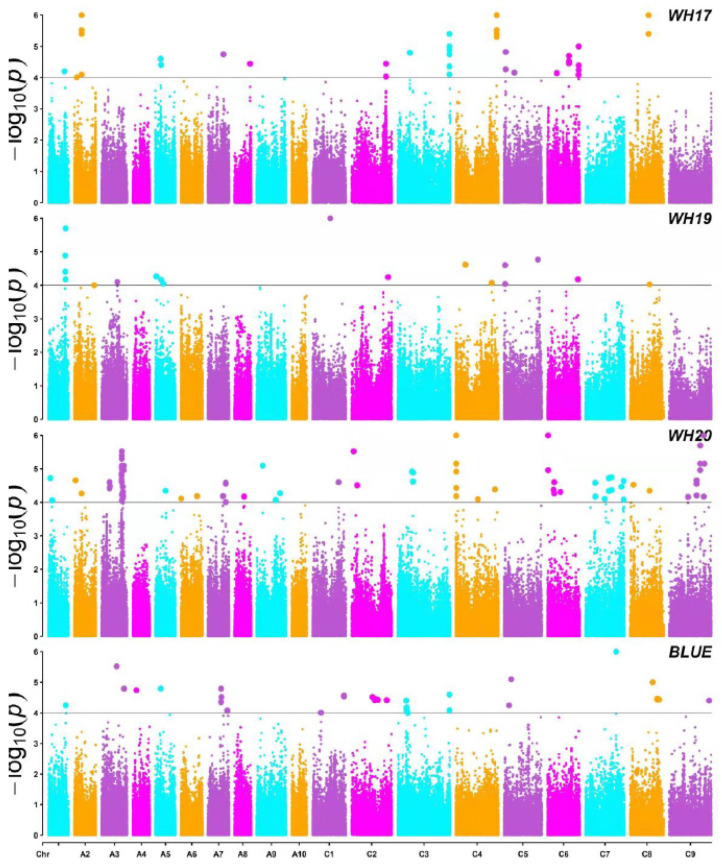
Manhattan plot of the phenotype-genotype association analysis for twelve root-related traits of *B. napus* by MLM in three environments and BLUE (WH17, WH19, WH20, and BLUE). The different colors in the plots indicate the 19 chromosomes from A01 to C09; the thick horizontal lines represent the threshold values (−log_10_1/20,131 = 4.30 × 10^−5^) 4.30 × 10^−5^. The color dots above the threshold values indicate the significant SNPs.

To dissect the genetic variations of mature root-related traits in *B. napus*, GWAS for these traits based on MLM with Q and K (Q + K) was conducted [38]. The significant SNPs associated with each trait were displayed on Manhattan plots (Figure 3; Figure S7A–D), and QQ plots (Figure S2) were generated. A total of 140 significant trait-SNP associations (−log_10_ (*p*) > 4.30, −log_10_1/20,131) for root-related traits were detected in the three environments, and 32 trait-SNP associations were detected using the best linear unbiased estimator (BLUE) values of the whole three environments. All the trait-SNP associations included 103 SNPs (Table S1). Except for Chromosomes A06 and A10, these SNPs were distributed across 17 Chromosomes of *B. napus*, and the phenotypic variance explained (PVE) values of these SNPs ranged from 5.24% (seq-new-rs38185) to 20.31% (seq-new-rs37128) (Table S1).

QTL clustering of correlated quantitative traits is a common phenomenon in plants [21,39]. The SNPs in the same haplotype block with high correlations may be clustered into the same QTL with pleiotropy. The haplotype blocks of all the significant SNPs were determined using the haploview software with the LD r^2^ > 0.2 between SNPs [38,40]. To further detect the effectiveness of a QTL related to root development, the SNPs in the same haplotype block could be repeatably detected for multiple traits or in different environments with suggestive associations (3.5 < −log_10_ (*p*) ≤ 4.30), and significant associations were integrated into a cluster. As a result, 128 significant and 76 suggestive trait-SNP associations, including 75 SNPs, with LD r^2^ > 0.2 and close vicinity (within 1 Mb; Liu et al. [38]) were clustered into 40 QTL clusters (Table S2), all of which included at least two investigated root-related traits. In addition, 20 pleiotropic QTL clusters with more than three root-associated traits were discovered, restating the previously mentioned trait correlation.

### 2.5. Important QTL Clusters for Root Development

Notably, four of the 40 QTL clusters, *qRT.A01-1, qRT.A02-1, qRT.A05-1*, and *qRT.C08-2*, were consistently detected in more than one environment (Table 3). Moreover, 11 pleiotropic loci, *qRT.A02-1, qRT.A05-2, qRT.A05-3, qRT.A09-2, qRT.A09-3, qRT.C03-2, qRT.C04-2, qRT.C04-3, qRT.C08-1, qRT.C09-1, and qRT.C09-4*, contributed more than 10% of the maximum PVE to the root traits (Table 3). These 14 important QTL clusters related to root traits may apply to improve RSA in rapeseed. Importantly, *qRT.A02-1*, both detected in WH17 and WH20, explained the maximum of 12.93% phenotypic variance to TSA and affected RFW, RDW, and almost all root morphological traits, except TRN0.5. Additionally, *qRT.A05-3* affected RFW, RDW, and all root morphologic traits displayed the highest PVE of 20.31%. In addition, *qRT.A05-2, qRT.C09-2, qRT.C03-2*, and *qRT.C04-2* influenced similar traits, TSA, TRL0.5, TSA0.5, and TRN0.5, and each contributed PVE of more than 13% to the traits. These traits are essential for RSA, because they increase the volume of the soil occupied by the root, allowing it to anchor and participate in water and nutrient uptake. The *qRT.C08-1* explained 14.24% of the trait variation and influenced several traits, including RFW and RDW. The *qRT.A09-2, qRT.C04-2*, and *qRT.C09-1* clusters are also linked to several root morphology-related traits involving TSA, TRV, and TRL, which could be exploited to increase nutrient acquisition and utilization efficiency in rapeseed, explaining a moderate PVE of more than 10%. Moreover, *qRT.C08-2*, stably identified in both WH19 and WH20, explained 6.28% PVE to PRL. *qRT.A01-1*, which was found to be stable in both WH17 and WH19, accounted for 14.73% of total phenotypic variance, and was related to TSA, TRL0.5, TSA0.5, and TRN0.5, which are targeted traits that may help improve rapeseed production.

### 2.6. Potential Candidate Genes Mining

To mine candidate genes related to root development, we retrieved all the genes in the 100 kb window (LD region) around each lead SNP within the 14 important QTL clusters. As a result, a total of 394 (Table S4) annotated gene models have been discovered according to the gene annotation of the *B. napus* ‘Damor’ genome. The gene number around the lead SNPs ranged from 26 to 72. The corresponding gene function was predicted based on the annotation details of the retrieved genes and the functions defined for their homologs in *A. thaliana* (Table S4). We created a protein interaction network from STRING (http://string-db.org/cgi/, accessed on 05 September 2021), employing all 396 genes in the LD region around each lead SNP within the 14 crucial QTL clusters to further investigate the genes functional interactions. There were 334 nodes and 260 edges in the network (Figure S3). A total of 334 GWAS candidate genes were marked with purple, green, red, and blue nodes in the network (Figure S5). As depicted in the interaction, *BnaC09g23910D*, *BnaC09g36350D*, *BnaC08g17600D*, *BnaC08g17710D*, *BnaA09g37460D*, *BnaA05g21560D, BnaC04g44940D*, *BnaC08g17760D*, and *BnaC04g47870D* all demonstrated significant interactions and may play essential roles via interacting with other associated genes.

It is possible that the gene *BnaC09g36350D*, in particular, will play a significant role in the networks. As previously stated, *HD2B*, the *Arabidopsis* homolog gene of *BnaC09g36350D*, is functional and required for proper root development [41]. This finding indicates that the main genes should be explored further to learn more about their potential roles in the network. 

The expression levels of all 396 genes in six different tissues and their Arabidopsis homolog genes in root tissues were checked using the *Brassica napus* Transcriptome Information Resource database (http://yanglab.hzau.edu.cn/BnTIR, accessed on 13 September 2021). Some genes’ expression was low to unobservable in the root tissue, indicating that they functioned as pseudogenes. Some were discovered to be significantly expressed in the cotyledon, root, leaf, silique, stem peel, and the seed of *B. napus*, implying that they are implicated in plant growth and development in the same way that they are in other plant species [42]. Genes with an expression level of log_2_^(TPM + 1)^ ≥ 10 [43] were considered candidate genes for root development. As a result, when compared to the previously reported root-related genes (Figure S5), the 32 novel potential genes, which have high expression in the root (Figure S4A,B), are involved in root growth and development, hormonal signaling pathways, root development, and abscisic acid-activated signaling pathtableways (Table 4). Among the 32 candidate genes, *BnaC09g36350D*, an essential candidate gene, also checked in the above protein interaction network, which was located on −16.806 kb downstream of the peak SNP Bn-scaff_17799_1-p298794 of *qRT.C09-4*, encodes *Arabodpsis thaliana’s histone deacetylase 2B* *(AtHD2B)*, which is functional and needed for normal root development [42]. Besides that, *BnaC03g28330D* is the Arabidopsis homolog of the *EXODIUM (EXO),* which is involved in a signaling mechanism that coordinates Brassinolide-responses with environmental or developmental stages and is located 8.273 kb upstream of the peak SNP Bn-scaff 15782 p111218 of the QTL cluster *qRT.C03-2* [44]. *BnaA05g21670D*, led by the SNP seq-new-rs37128 of *qRT.A05-3*, is the Arabidopsis homolog of *ASPARTIC PROTEASE IN GUARD CELL 1 (ASPG1)*, which modulates gibberellic acid signaling by degrading hormonal transcriptional regulators [45]. *BnaC09g36350D*, the putative candidate gene discovered in *qRT.C09-4*, is homologous to Arabidopsis *Histone deacetylase 2B* (*HD2B)*. *AtHD2C* and *AtHD2B* restored the leaf and root developmental abnormalities of *hd2b* and *hd2c* to respective single mutants [42], implying that *HD2C* and *HD2B* are functional and essential for optimal leaf and root development. The detected SNPs and potential candidate genes will be helpful for prospective functional characterization of rapeseed to improve RSA.

### 2.7. Phylogenetic Trees, Gene Structure Analysis, and Subcellular Localization Prediction

**Figure 4:**
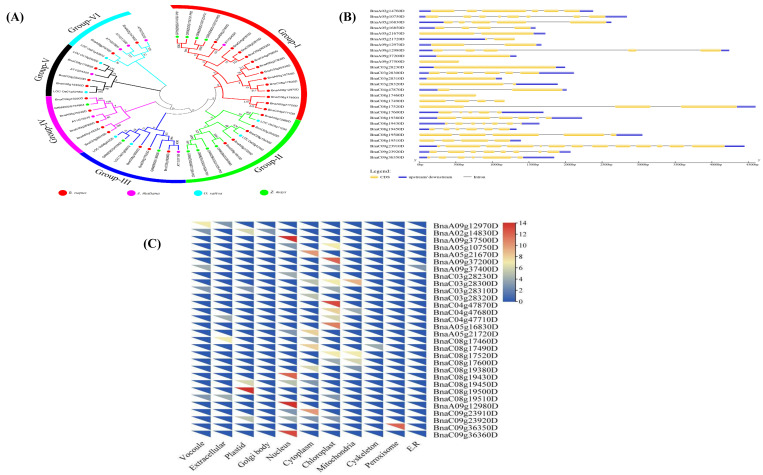
Phylogenetic tree analysis, gene structure, and subcellular localization of the *B. napus* candidate genes (**A**) Phylogenetic tree and subgroup representation of genes in *B. napus, A. thaliana, Zea mays,* and *Oryza sativa*. MEGA 7 software was used to construct the phylogenetic tree using the neighbor-joining method with a bootstrap value of 1000. The numbers beside the branches show the bootstrap values. (**B**) The distribution of exons and introns in the genes. (**C**) The distribution of genes throughout subcellular organelles. The red and yellow hue shows the density of the gene in the organelle.

Phylogenetic analysis of these genes from *B. napus, A. thaliana, Zea mays,* and *Oryza sativa* was used to categorize the identified 32 potential genes. The genes were divided into six groups, referred to as Groups I through VI. With 18 members, Group-I was the most populous, while Group-V and Group-VI each had only six (Figure 4A). Thirteen of the 32 genes were discovered in Group-I, with only two in Group-VI. Genes from rapeseed and three other plant species, including *Brassica rapa*, *Brassica oleracea*, and *Arabidopsis thaliana*, were classified into four main groups by Li et al. [46]. The coding sequences of the putative genes were aligned to the genomic sequences to explore gene structure evolution further and examine structural features. All of the genes’ protein lengths differed, demonstrating that the differences in their gene structures are not only attributable to differences in intron numbers and sizes (Figure 4B). Exon numbers were also lost and gained during evolution, indicating functional variability among the closely related genes [47]. Four genes do not have introns according to our findings. Thirteen of the candidate genes were found to be localized in the chloroplast, ten in the nucleus, ten in the mitochondria, and one each in the cytoskeleton, peroxisomes, Golgi body, and endoplasmic reticulum, respectively. Each of the two genes was found in three separate subcellular locations. These findings show that these genes in *B. napus* have a wide range of functions (Figure 4C).

### 2.8. GO and KEGG Analysis of Root-Related Traits Genes

To learn more about the function of the candidate genes, we used GO enrichment analysis and KEGG pathways analysis on the 32 candidates (Tables S7 and S8). They were well-represented in the three GO classes of biological process, cellular component, and molecular function. Figure S6A&B show the most commonly used GO terms (according to their *p*-values). The most common GO terms in the biological process category were the dTDP-rhamnose biosynthetic process, selenium compound metabolic process, and arginine catabolic process. The most common GO terms in the molecular function category were dTDP-4-dehydrorhamnose reductase activity and dTDP-4-dehydrorhamnose 3,5-epimerase activity. In the cellular component category, the most common GO terms were cell wall, eukaryotic translation initiation factor 3 complex, eIF3e, and multi-eIF complex. Remarkably, some genes were classified into many categories. We employed KEGG enrichment analysis to understand these genes’ metabolic pathways (Figure S6C). Phosphonates and phosphinates metabolism (bna00440), vitamin B6 metabolism (bna00750), monobactam biosynthesis (bna00261), and selenocompound metabolism (bna00450) were all shown to be substantially enriched.

According to the KEGG pathway analysis, *BnaC08g17490D*, the Arabidopsis homolog of pyridoxal phosphate phosphatase-related protein, is involved in vitamin B6 metabolism. The short root phenotype of *rsr4-1* was followed by the production of lateral roots early in Arabidopsis, predominantly at the base of the hypocotyl. With the lack of vitamin B6, lateral roots began closer together than in the wild type, according to Wanger et al. [48]. In addition, the Arabidopsis homolog of *ATP sulfurylase 1 (APS1)*, *BnaA05g16830D*, has a role in selenocompound metabolism. Because of the loss of cell viability in the root apex, Lehotai et al. [49] noticed significant changes in the root architecture of the selenite-treated plants.

## 3. Discussion

Rapeseed root architecture varies substantially depending on development patterns; the winter canola’s root system is more potent and broader than the root system of spring rapeseed [23]. Three subpopulations were investigated for genetic variation in root architecture traits in this study. This diversity collection revealed subpopulations differentiated mainly through their growth habits, which corresponded to the results reported by others [50,51]. The breeding history of winter, semi-winter, and spring rapeseed lines was assigned to distinct groups. A genetic process controls the need for vernalization to stimulate the commencement of flowering, which controls the differentiation into winter, semi-winter, and spring forms [52]. In comparison to the winter and semi-winter type lines, spring rapeseed lines had the longest primary length (Figure S8). These results supported a recent greenhouse study that found the primary growth of spring rapeseed lines were fast when compared to winter and swede-type lines [53]. The primary and lateral roots of winter rapeseed and fodder were longer than spring rapeseed, according to Thomas et al. [54]. Zhang et al. [28], on the other hand, suggested that lower PRL will lead to higher LRD because the latter is inversely proportional to the former. As a result of this research, it was discovered that genotype had a major impact on root growth dynamics.

The overlapping of QTL for root characteristics with productivity (yield, water usage, or nutrient acquire) has indicated that the former may play a role in deciding the latter in many cases [55]. Furthermore, the rising amount of available data for specific QTL extends our physiological and evolutionary comprehension, revealing similarities among root morphology and root functions, which will be critical in developing RSA for specific environments. For example, the QTL *DRO1* controlling both root depth and root growth angle in rice has been deployed to improve RSA for the high water effectiveness of an Indian upland rice variety by marker-assisted selection [55]. Thus, the quest for QTL has been a practical research approach in investigating RSA’s genetic variation. 

The GWAS method is widely acknowledged as a powerful technique for connecting phenotypes to their underpinning genetics, with more precision than traditional linkage mapping [56]. Previously, researchers used the linkage mapping method to investigate the genetics of root-related traits [21,26,57,58]. When we compared our findings to earlier QTLs, we discovered multiple overlapping loci linked to similar traits. Three, two, and one significant QTLs found in different environments by Wang et al. (2017), Dun et al. [57], and Shi et al. [27] matched significant loci found in our study (Table S4), suggesting that these trait associations are inherently stable and could be very beneficial to expedite rapeseed RSA continuous improvement. The results of our GWAS identified some clusters of significant loci, emphasizing key genetic regions linked to root-related traits. Surprisingly, some pleiotropic QTLs were discovered and related to the same traits (Table S4). These findings show that distinct root-related traits from similar QTLS have similar genetic architecture. Therefore, different traits from specific QTLs can be treated independently to improve RSA in rapeseed. Similarly, Zhang et al. [28] discovered overlapping loci for several root-related traits in *Brassica napus*.

However, transcriptome analysis is commonly used to assess gene expression changes, allowing for more efficient and accurate candidate gene finding in GWAS [59]. Numerous potential genes were obtained from genomic regions closely related to the traits studied in this research. Additional RNA-seq data could also be used to uncover the underlying genes for root-related traits, as shown in *Brassica napus*, rice, barley, maize, and other crops [8,21,60,61].

In breeding programs, associations with stronger phenotypic effects for desired traits are often more advantageous [62]. In some species with simplified genetic backgrounds, favorable alleles and unfavorable alleles can be identified without factoring in heterozygous SNPs [36,63]. At marker seq-new-rs37128, the proportion RFW, TRL, TSA, TRN, TRV, TRL0.5, TSA0.5, and TRN0.5 of accessions with favorable alleles (GG; N = 265) was 78.40% each, and that of RDW (GG; N = 218) was 64.50%, which is higher than the 19.53% and 15.38% of accessions with unfavorable alleles (GC; N = 65) and (GC; N = 52); and the proportion TSA, TRL0.5, TSA0.5, TRV0.5, and TRN0.5 of accessions with favorable alleles (GG; N = 265) at marker Bn-scaff 16888 1-p45860 were each 86.39%, which was greater than 9.47% for accessions with unfavorable alleles (AA; N = 32). Therefore, we classified “favorable alleles” as SNP alleles with greater of these alleles that promote root growth, and “unfavorable alleles” were defined as SNP alleles with lower alleles, including heterozygous sites as reported by [62]. These findings demonstrated that in *B. napus*, the genetic regulation of root growth has a mostly additive effect.

## 4. Materials and Methods

### 4.1. Plant Materials and Field Experiments

In this study, the association population consisted of 338 *B. napus* genotypes, including winter, semi-winter, and spring accessions obtained from breeding institutes based on the Rapeseed Research Network in China. There were 253 accessions from the Yangtze River of China, 37 from northwestern China, 21 from Europe, 19 from Australia, and 8 from other places or unknown origins. Accessions usually grow under the winter-growth conditions in China according to growth habits.

All 338 accessions were planted for three years in Wuhan (2016–2017, 2018–2019, and 2019–2020, designated as WH17, WH19, and WH20, respectively). For each accession, the self-pollinated seeds were planted in the seed with 10 cm for spacing in the rows and 33 cm for spacing between rows, and a randomized complete block design was followed with triplicates. At the end of September, each plot containing three rows was sown and harvested the following May.

### 4.2. Phenotypic Evaluation of the Association Panel

To identify the genetic mechanism of rapeseed root variation in the field, root-related traits of 338 natural accessions in the *B. napus* association panel were investigated under the mature stage within three environments, WH17, WH19, and WH20, respectively. By digging roots in the field at a mature stage, seven to eight uniform plants from each plot were sampled. Sampling was done after rain to ensure root integrity. Manually evaluated traits, including primary root length (PRL), root fresh weight (RFW), and diameter of root (DMR), were recorded once the plants had been sampled. Using WinRHIZO-Pro software (Regent Instruments, QC Quebec City, Canada) to determine total root length (TRL), total root volume (TRV), total root surface area (TSA), and total root number (TRN), the intact roots were scanned and analyzed. TRL0.5, TSA0.5, TRV0.5, and TRN0.5, defined as TRL, TSA, TRV, and TRN of roots with a diameter above 0.5 cm (Table 1). There were two statistical ways for root morphological traits captured from the WinRHIZO-Pro software: roots calculated for both the primary root and all lateral roots, including TSA, TRL, TRV, and TRN, and roots calculated for the primary root and the lateral roots with the diameter above 0.5 mm, including TSA0.5, TRL0.5, TRV0.5, and TRN0.5 because lateral roots with diameter less than 0.5 cm were easy to be damaged during the sampling process. 

### 4.3. Data Analysis

The mean values of each genotype were used for statistical analysis, and the data were analyzed by analysis of variance (ANOVA) based on the generalized linear model (GLM). Pearson’s method using the OriginPro software package (OriginLab Corporation, Northampton, MA, USA) was used to estimate the correlation between the root-related traits at a significant level of (*p* < 0.05) and the best linear unbiased estimator (BLUE) values of phenotypic data from the three environments were utilized for the correlation analysis. The broad-sense heritability (H^2^) was determined for each trait [64] as follows: H^2^ = (σ^2^G)/(σ^2^P), σ^2^G = (MSG−MSE/rep), σ^2^P = (MSG−MSE/rep) + MSE; H^2^ = (MSG−MSE/rep)/(MSG−MSE/rep) + MSE, where σ^2^G and σ^2^P are the genotypic and phenotypic variances, respectively, MSG and MSE represent the mean square of genotype and mean square error, respectively, estimated by analysis of variances (ANOVA) using SAS 9.3 (SAS Institute Inc., Cary, NC, USA), and rep is the number of replications.

### 4.4. SNP Genotyping and Marker Filtering

The genomic DNA of the 338 *B. napus* lines has been extracted using the CTAB method from young leaves. To genotype the association population, a new *B. napus* 50K Illumina Infinium SNP array developed by Greenfafa Biotech Co., Ltd. (Wuhan, China) was used with 45,707 SNPs. The SNP data were first clustered and automatically called by blasting against the ‘Damor’ genome [65] using Genome Studio software (Illumina, Inc., San Diego, CA, USA). The probe sequences of these SNPs with an e-value threshold of e^−10^ were remained for further analysis, excluding SNPs that matched two or more locations with the same top e-values. A technique known as by-filtering analysis has been designed to increase the performance and quality of SNP array data analysis [66]. After bi-filtering analysis in the natural population with missing rate ≤0.2, heterozygous rate ≤0.2, and minor allele frequency >0.05, 20,131 SNPs remained for further analysis.

### 4.5. Population Structure, Relative Kinship, and LD Analysis

Population structure and the relative kinship of the 338 *B. napus* accessions were computed using STRUCTURE v. 2.3.4 and SPAGeDi software, respectively [67]. All negative values were set to zero between the two accessions. TASSEL 5.0 [68] was used to test the linkage disequilibrium (LD) decay by the parameter r^2^ among all SNPs [69]. Marker haplotypes were established for each associated locus using the haploview software as previously reported [70].

### 4.6. Genome-Wide Association Analysis

Based on 20,131 SNP markers selected in this study, genome-wide association analysis (GWAS) for the root-related traits was performed by the mixed linear model (MLM) with (Q + K) matrix using the Tassel 5.0 software [68]. The arbitrary threshold value was set as 1/20,131 SNPs (−log_10_ (*p*) = 4.30) to identify the marker-trait associations. The Manhattan plot and Quantile-Quantile plot (Q-Q plot) were drawn by qqman [71] and ggplot2 software [72]. By comparing *p* values with the arbitrary threshold (1/20,131 = 4.30 × 10^−5^), significant statistical loci were detected.

### 4.7. Determination of Candidate Genes

Potential candidate genes within the detected loci were searched within 100 kb upstream and downstream of the significant lead SNPs linked to each trait. The *B. napus* reference genome information [65] was used to scan the entire gene list in the QTL region, and potential candidate genes linked to root growth and development were determined according to the Gene Ontology terms (GO terms) from the TAIR website and gene functions retrieved from previous studies.

### 4.8. Protein Interaction Network Analysis, Phylogenetic Trees, Gene Structure Analysis, and Subcellular Localization Prediction

We used the internet program STRING (http://string-db.org/cgi/, accessed on 05 September 2021) to build a protein interaction network with all the GWAS genes obtained from the LD region around each lead SNP to further study the gene’s functional relationships. To conduct the gene structure analysis, GSDS (http://gsds.cbi.pku.edu.cn, accessed on 20 September 2021) was used. From the respective plant databases, the protein sequences of various plant homologous genes were retrieved. Sequence alignment was analyzed using ClustalX software version 1.2 [73]. Using the bootstrap method with 1000 replications based on protein sequences with MEGA 7.0 software, an NJ phylogenetic tree has been constructed. Protein sequences of the candidate genes were utilized to estimate subcellular localization using the online tool Wolf PSORT (https://wolfpsort.hgc.jp, accessed on 20 September 2021) for predicting subcellular protein localization.

## 5. Conclusions

The genetic dissection of *B. napus* root-related traits at the mature stage in the field in three different environments was investigated in this study using GWAS with MLM (Q + K) model analysis. In the three environments and BLUE, 172 marker-trait associations for root-related traits were identified on all chromosomes except A06 and A10. The genetic regions that influence root development were found to be primarily on the A03 and C08 chromosomes. We discovered 14 key QTL clusters associated with root traits that could be leveraged to improve RSA in rapeseed. Five of these loci were found to be overlapped with the previously reported QTLs. Using the *Brassica napus* Transcriptome Information Resource database (http://yanglab.hzau.edu.cn/BnTIR, accessed on 13 September 2021), 32 orthologs of functional candidate genes related to root development were identified in a distance of 100 kb around these significantly marker-trait associations of the 40 key QTL clusters, based on the expression levels of all 396 genes in six different tissues, as well as their Arabidopsis homolog genes in root tissues. These significant QTL clusters and candidate genes may give new sources for molecular breeding and functional study of rapeseed root growth and development. The procedure of identifying causative genes is quite difficult. More research is required to determine the molecular roles of these potential genes by more detailed examinations. Nonetheless, integrating GWAS and transcriptome analysis to identify candidate genes efficiently could be a useful technique for unraveling the quantitative genes implicated in *B. napus* root development.

## Figures and Tables

**Table 1:** Summary of the 12 evaluated root-related traits and their descriptions.

Traits	Abbreviation	Unit
Primary root length	PRL	cm
Diameter of root	DMR	cm
Root fresh weight	RFW	g
Root dry weight	RDW	g
Total root length	TRL	cm
Total root surface area	TSA	cm^2^
Total root volume	TRV	cm^3^
Total number of roots	TRN	N
Total root length above 0.5 cm	TRL0.5	cm
Total root surface area above 0.5 cm	TSA0.5	cm^2^
Total root volume above 0.5 cm	TRV0.5	cm^3^
Total number of the root above 0.5 cm	TRN0.5	N

**Table 2:** Phenotypic variations for root-related traits of 338 *B. napus* genotypes.

Trait	Environment	Mean	SD	Min	Max	CV (%)	H^2^ (%)
PRL (cm)	WH17	20.25	3.13	11.98	30.04	15.44	66.47
	WH19	21.17	2.19	16.23	28.19	10.33	
	WH20	26.49	2.48	17.31	32.34	9.36	
DMR (cm)	WH17	15.18	2.61	9.00	24.83	17.19	59.19
	WH19	14.10	1.54	10.97	19.51	10.93	
	WH20	13.30	1.70	9.04	20.92	12.78	
RFW (cm)	WH17	23.56	6.68	12.51	71.18	28.34	59.79
	WH19	18.83	4.40	8.67	32.84	23.35	
	WH20	19.17	5.15	7.01	36.65	26.88	
RDW(g)	WH17	4.38	1.44	1.68	13.45	32.93	66.58
	WH19	NA	NA	NA	NA	NA	
	WH20	NA	NA	NA	NA	NA	
TRL (cm)	WH17	1269.9	497.1	297.4	4316.1	39.14	79.68
	WH19	535.9	219.7	138.7	1423.7	40.99	
	WH20	342.5	147.9	94.3	1231.1	43.19	
TSA (cm^2^)	WH17	217.6	62.5	73.1	687.5	28.73	72.88
	WH19	137.8	31.6	58.9	241.4	22.96	
	WH20	117.9	27.5	58.8	270.0	23.28	
TRV (cm^3^)	WH17	3.07	0.82	1.14	9.16	26.65	62.57
	WH19	3.35	0.94	1.85	9.86	28.11	
	WH20	3.71	1.02	2.01	7.52	27.60	
TRN (N)	WH17	3387.1	1523.7	608.1	12,153.6	44.99	72.73
	WH19	3903.0	1886.0	978.0	12,868.0	48.32	
	WH20	3846.0	2596.0	420.0	23,986.0	67.50	
TRL0.5 (cm)	WH17	240.3	73.3	59.1	687.2	30.52	74.83
	WH19	107.4	33.4	38.3	215.3	31.13	
	WH20	97.1	28.4	41.3	287.5	29.29	
TSA0.5 (cm^2^)	WH17	130.2	35.4	46.4	400.1	27.21	66.18
	WH19	85.4	18.7	39.7	138.4	21.91	
	WH20	90.8	18.9	44.0	208.3	20.79	
TRV0.5 (cm^3^)	WH17	20.04	6.63	5.40	65.20	33.06	55.18
	WH19	18.67	3.95	8.51	32.31	21.14	
	WH20	19.74	4.81	10.18	35.83	24.37	
TRN0.5 (N)	WH17	44.90	12.80	16.27	99.10	28.52	67.06
	WH19	27.65	7.96	12.67	67.60	28.79	
	WH20	21.56	6.43	8.92	52.01	29.83	

**Table 3:** Information of crucial QTL clusters related to root traits.

Locus	Chr.	Peak SNP	Position of Peak SNP	Number of SNP-Trait Associations	Trait	−log10(P)	PVE (%)	Haplotype Block (Mb)
qRT.A01-1	A01	seq-new-rs27069	19833111	4	WH19-TRV, WH17-TRN	4.88	7.71	19.280–19.867
*qRT.A02-1*	A02	Bn-A02-p11772983	8480672	10	WH17-(RFW, RDW, TRL, TSA, TRV, TRN, TRL0.5, TRV0.5, TSA0.5), WH20-RFW	7.80	12.93	8.451–8.705
*qRT.A05-1*	A05	Bn-A05-p6415983	5964030	7	WH17-(RFW, TSA, TRV0.5), WH19-PRL, BLUE-RDW	4.81	7.13	5.959–6.458
*qRT.A05-2*	A05	Bn-A05-p15113541	11594569	4	WH20-(TSA, TRL0.5, TSA0.5, TRN0.5)	8.97	13.32	11.340–11.862
*qRT.A05-3*	A05	seq-new-rs37128	16647352	11	WH17-(RFW, RDW, TRL, TSA, TRV, TRN, TRL0.5, TSA0.5, TRN0.5), BLUE-(RFW, RDW)	11.91	20.31	-
*qRT.A09-2*	A09	seq-new-rs41374	7034148	3	WH20-(TRL, TSA, TRN)	6.49	10.44	-
*qRT.A09-3*	A09	seq-new-rs48456	26867775	4	WH20-(TSA, TRL0.5, TSA0.5, TRN0.5)	9.83	14.73	26.643–26.926
*qRT.C03-2*	C03	Bn-scaff_15782_1-p111218	16702113	4	WH20-(TSA, TRL0.5, TSA0.5, TRN0.5)	9.31	13.81	16.684–16.902
*qRT.C04-2*	C04	Bn-scaff_16888_1-p45860	44839791	5	WH20-(TSA, TRL0.5, TSA0.5, TRV0.5, TRN0.5)	9.20	13.94	44.773–45.195
*qRT.C04-3*	C04	Bn-scaff_20270_1-p620404	46737401	5	WH17-(TSA, TRV, TRL0.5, TSA0.5, TRV0.5)	6.19	10.25	46.605–46.986
*qRT.C08-1*	C08	C08_21120661	21120661	9	WH17-(RFW, RDW, TRL, TSA, TRN, TRL0.5, TSA0.5, TRV0.5)	8.62	14.24	20.814–21.123
*qRT.C08-2*	C08	seq-new-rs39808	22313636	2	WH19-PRL, WH20-PRL	4.35	6.28	-
*qRT.C09-1*	C09	seq-new-rs36496	21582357	5	WH20-(TRL, TSA, TRL0.5, TSA0.5, TRN0.5)	7.49	10.99	21.573–21.927
*qRT.C09-4*	C09	Bn-scaff_17799_1-p2987946	39625508	4	WH20-(TSA, TRL0.5, TSA0.5, TRN0.5)	9.11	13.66	39.357–39.838

**Table 4:** Candidate genes for root-related traits within 100 kb upstream and downstream of the lead SNPs.

Locus	Candidate Gene	Distance to Peak SNP (kb)	Homologous Genes in At	Gene Symbol	Description
*qRT.A02-1*	BnaA02g14760D	60.725	AT1G70160.1	*-*	-
	BnaA02g14780D	28.280	AT1G70260.1	*RTP1*	RESISTANCETOPHYTOPHTHORA PARASITICA 1
	BnaA02g14830D	−25.412	AT1G70330.1	*ENT1*	Equilibrative nucleotide transporter 1
*qRT.A05-1*	BnaA05g16830D	62.513	AT3G22890.1	*APS1*	ATP sulfurylase 1
	BnaA05g10750D	97.767	AT2G32260.1	*CCT1*	Phosphorylcholine cytidylyltransferase
*qRT.A05-3*	BnaA05g21720D	−48.519	AT3G18410.2	*NDUFS6*	Complex I subunit NDUFS6
	BnaA05g21670D	−17.913	AT3G18490.1	*ASPG1*	ASPARTIC PROTEASE IN GUARD CELL 1
*qRT.A05-2*	BnaA05g16850D	10.872	AT3G22845.1	*-*	Emp24/gp25L/p24 family/GOLD family protein
*qRT.A09-2*	BnaA09g12980D	17.222	AT1G62990.1	*KNAT7*	KNOTTED-like homeobox of Arabidopsis thaliana 7
	BnaA09g12970D	22.187	AT1G63000.1	*NRS/ER*	nucleotide-rhamnose synthase/epimerase-reductase
*qRT.A09-3*	BnaA09g37200D	80.012	AT3G57990.1	*-*	-
	BnaA09g37400D	−43.113	AT3G58460.1	*RBL15*	RHOMBOID-like protein 15
	BnaA09g37500D	−94.027	AT4G18610.1	*LSH9*	LIGHT SENSITIVE HYPOCOTYLS 9
*qRT.C03-2*	BnaC03g28230D	68.483	AT4G08810.1	*-*	SUB1
	BnaC03g28300D	28.483	AT4G08900.1	*-*	Arginase
	BnaC03g28310D	12.796	AT4G08950.1	*EXO*	EXORDIUM
	BnaC03g28320D	−1.474	AT4G08960.1	*PTPA*	phosphotyrosyl phosphatase activator (PTPA) family protein
*qRT.C04-3*	BnaC04g47870D	−21.135	AT1G58684.1	*-*	Ribosomal protein S5 family protein
	BnaC04g47680D	87.509	AT2G41835.1	*ERD15*	EARLY RESPONSIVE TO DEHYDRATION 15
	BnaC04g47710D	77.159	AT2G41475.1	*ATS3*	Embryo-specific protein 3
*qRT.C08-1*	BnaC08g17460D	36.663	AT1G17620.1	*LEA*	Late embryogenesis abundant (LEA) hydroxyproline-rich glycoprotein family
	BnaC08g17490D	21.642	AT1G17710.1	*-*	Pyridoxal phosphate phosphatase-related protein
	BnaC08g17520D	80.800	AT1G17745.1	*PGDH*	3-phosphoglycerate dehydrogenase (PGDH)
	BnaC08g17600D	28.805	AT1G17880.1	*BTF3*	Basic transcription factor 3
*qRT.C08-2*	BnaC08g19380D	7.866	AT1G20650.1	*-*	Protein kinase superfamily protein;
	BnaC08g19430D	−16.450	AT1G20696.2	*HMGB3*	High mobility group B3
	BnaC08g19450D	−23.572	AT1G20770.1	*SAY1*	*-*
	BnaC08g19500D	−71.232	AT1G20840.1	*TMT1*	Tonoplast monosaccharide transporter1
	BnaC08g19510D	−78.520	AT1G20850.1	*XCP2*	Xylem cysteine peptidase 2
*qRT.C09-1*	BnaC09g23910D	−77.343	AT4G11420.1	*EIF3A*	Eukaryotic translation initiation factor 3A
*qRT.C09-4*	BnaC09g36350D	−16.806	AT5G22650.2	*HD2B*	Histone deacetylase 2B
	BnaC09g36360D	−19.204	AT5G22640.1	*Emb1211*	Embryo defective 1211

## Data Availability

The datasets generated during and/or analyzed during the current study are available from the corresponding author on reasonable request.

## Supplementary Materials

The following are available online at https://www.mdpi.com/article/10.3390/plants10122569/s1; Supplementary File S1: Table S1: Summary of significant SNPs associated with root-related traits in *B. napus* detected in WH17, WH1,9, WH20, and BLUE; Table S2: Summary of QTL clusters associated with root-related traits in *B. napus* detected in WH17, WH19, WH20, and BLUE; Table S3: List of all potential candidate genes within 100 kb upstream and downstream of the lead SNPs; Table S4: Comparison of common QTLs detected for root-related traits between our GWAS results and previous linkage mapping QTLs; Table S5: Phenotypic variations of the examined 12 traits in Brassica over a course of three years; Table S6. Summary of SNP and linkage disequilibrium (LD) decay on the 19 chromosomes of *B. napus*; Table S7: GO enrichment results of the candidate genes; Table S8: KEGG enrichment results of the candidate genes; Table S9: Correlation analyses between root-related traits across the three environments (WH17, WH19, and WH20). Supplementary File S2: Figure S1. Distribution of the evaluated root-related traits in the association population of 327 *B. napus* grown in three environments (WH17, WH19, and WH20). The fitted are in red, green, and blue. Refer to Table 1 for the definition of terms; Figure S2. Quantile-quantile plots of estimated-log10(P) from phenotype-genotype association analysis of twelve root-related traits using the MLM model in three environments and BLUE (WH17, WH19, WH20, and BLUE); Figure S3. Network of protein interactions. The gene connections were suggested by the network. Proteins are represented by network nodes, while query proteins and the initial shell of an interactor are represented by colored nodes; Figure S4. Expression profile of candidate genes. (A) *Brassica napus* expression profiles of potential genes in six distinct tissues. (B) Arabidopsis homologous gene expression profiles. The heat map is based on the log2(TPM + 1) values. The redder the color, the more linked genes are overexpressed. Genes are grouped via hierarchical clustering; Figure S5. Distribution of our and previously reported candidate genes for root growth and development across 18 of *Brassica napus*’ 19 chromosomes. Our candidate genes were indicated by black boxes; Gray, Arifuzzaman et al., 2020b; Blue, Zhang et al., 2015; Green, He et al., 2019; Red, Fletcher et al., 2016; Purple, Arifuzzaman et al., 2019; Orange, Arifuzzaman et al., 2020a; Yellow, Duan et al., 2021, Pink, Wang et al., 2017; Gold, Li et al., 2021; Dark red, Wang et al., 2019. Supplementary File S3: Figure S6. Functional annotation of the detected candidate genes. (A) GO classification of the genes (B) GO analysis of the top 30 genes (C) KEGG analysis of the top 30 genes. Supplementary File S4: Figure S7A. Manhattan of phenotype-genotype association analysis for twelve root-related traits of *B. napus* in each environment and the BLUE. Supplementary File S5: Figure S8. The diversity collection of *Brassica napus* revealed distribution in three subpopulations: winter rapeseed, spring rapeseed, and semi-winter rapeseed. The subpopulations are represented by the three colors of the bars.

## Abbreviations

GWAS, genome-wide association study; QTL, quantitative trait loci; MLM, mixed linear model; GLM, generalized linear model; SNP, single nucleotide polymorphism; LD, linkage disequilibrium; RSA, root system architecture; qRT, cluster of root trait; PVE, phenotypic variance explained; Q-Q plot, quantile-quantile plot; MAF, minor allele frequency.
