# Supplementary material for: Genetic Dissection of Mature Root Characteristics by Genome-Wide Association Studies in Rapeseed (Brassica napus L.)
Source: Plants (Basel). 2021 Nov 24;10(12):2569. doi: 10.3390/plants10122569 (PMC8705616; doi:10.3390/plants10122569)
Supplement: Supplementary file 1 [file plants-10-02569-s001.zip › Supplementary file 2.pdf]

Figure S1

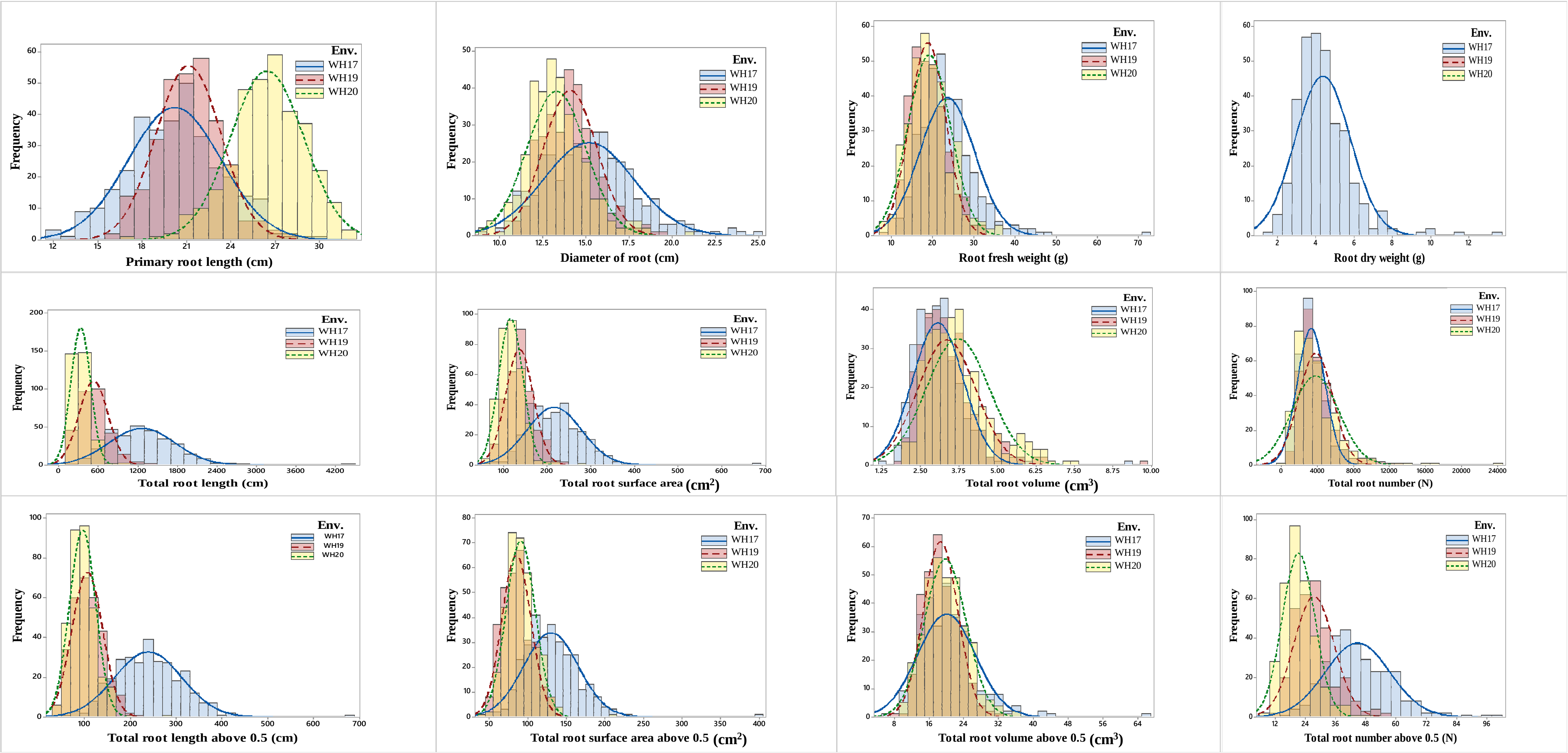

Distribution of the evaluated root-related traits in the association population of 327 *B. napus* grown in three environments (WH17, WH19, and WH20). Fitted curves are in red, green, and blue. Refer to table 1 for the definition of terms.

Figure S2

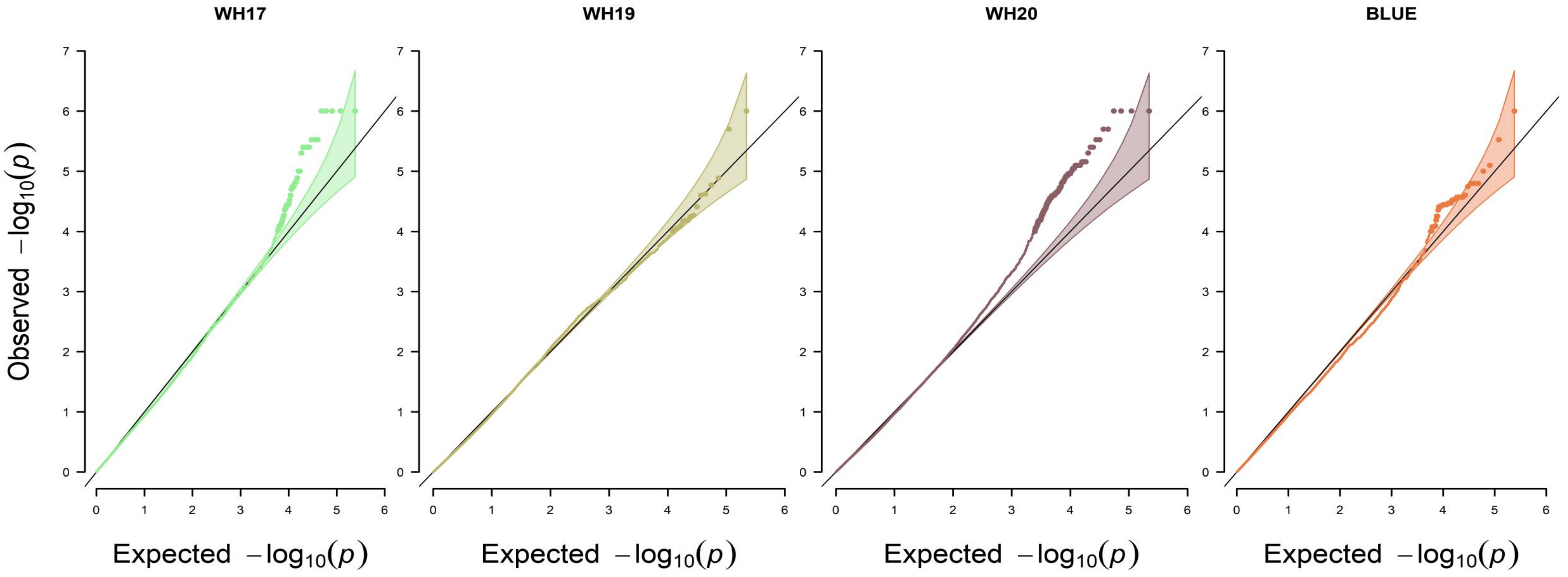

Quantile-quantile plots of estimated-$\log_{10}$(P) from Phenotype-genotype association analysis of twelve root-related traits using MLM model in three environments and BLUE (WH17, WH19, WH20, and BLUE).

Figure S3

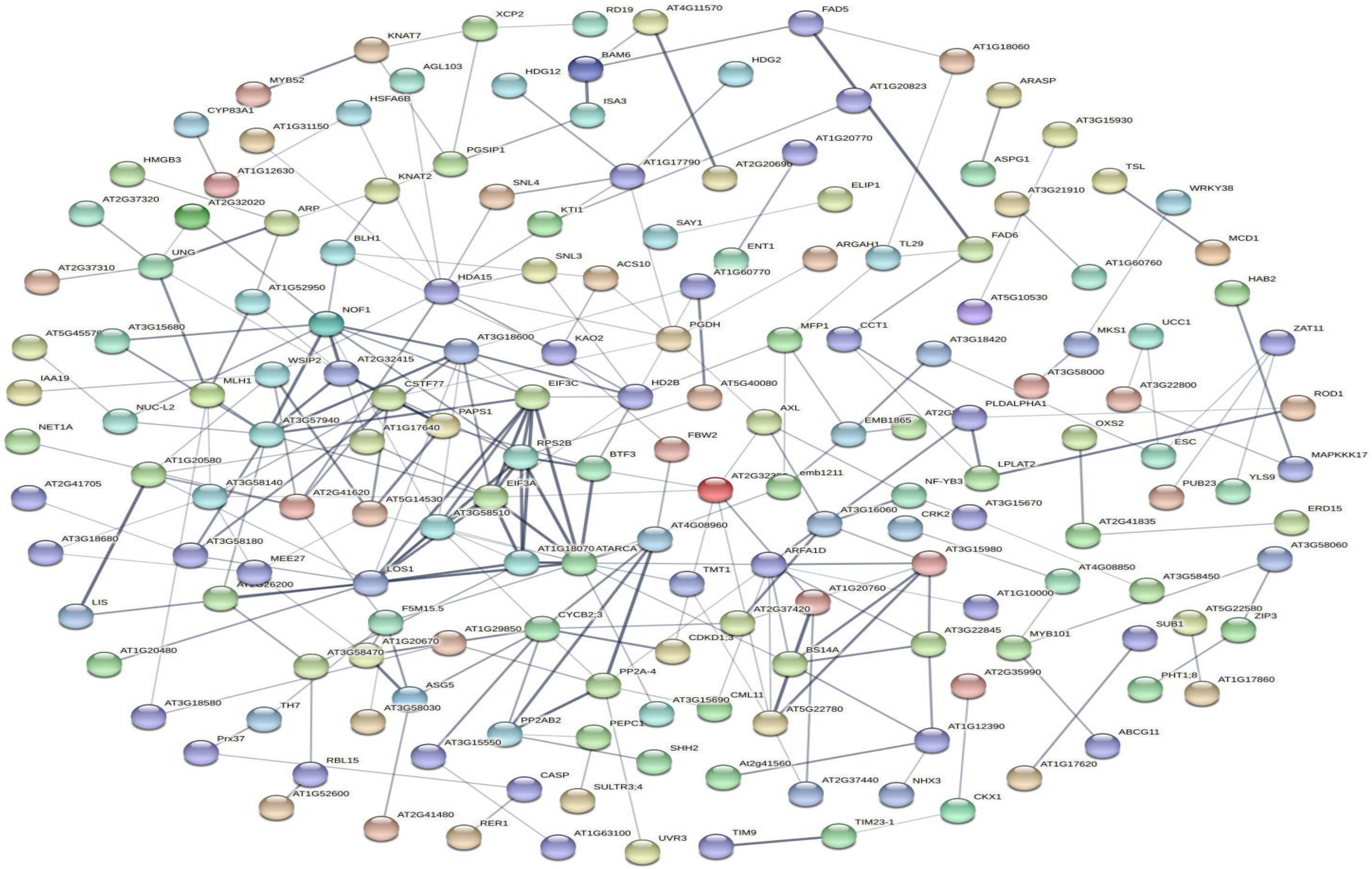

Network of protein interactions. The gene connections were suggested by the network. Proteins are represented by network nodes, while query proteins and the initial shell of an interactor are represented by colored nodes.

Figure S4

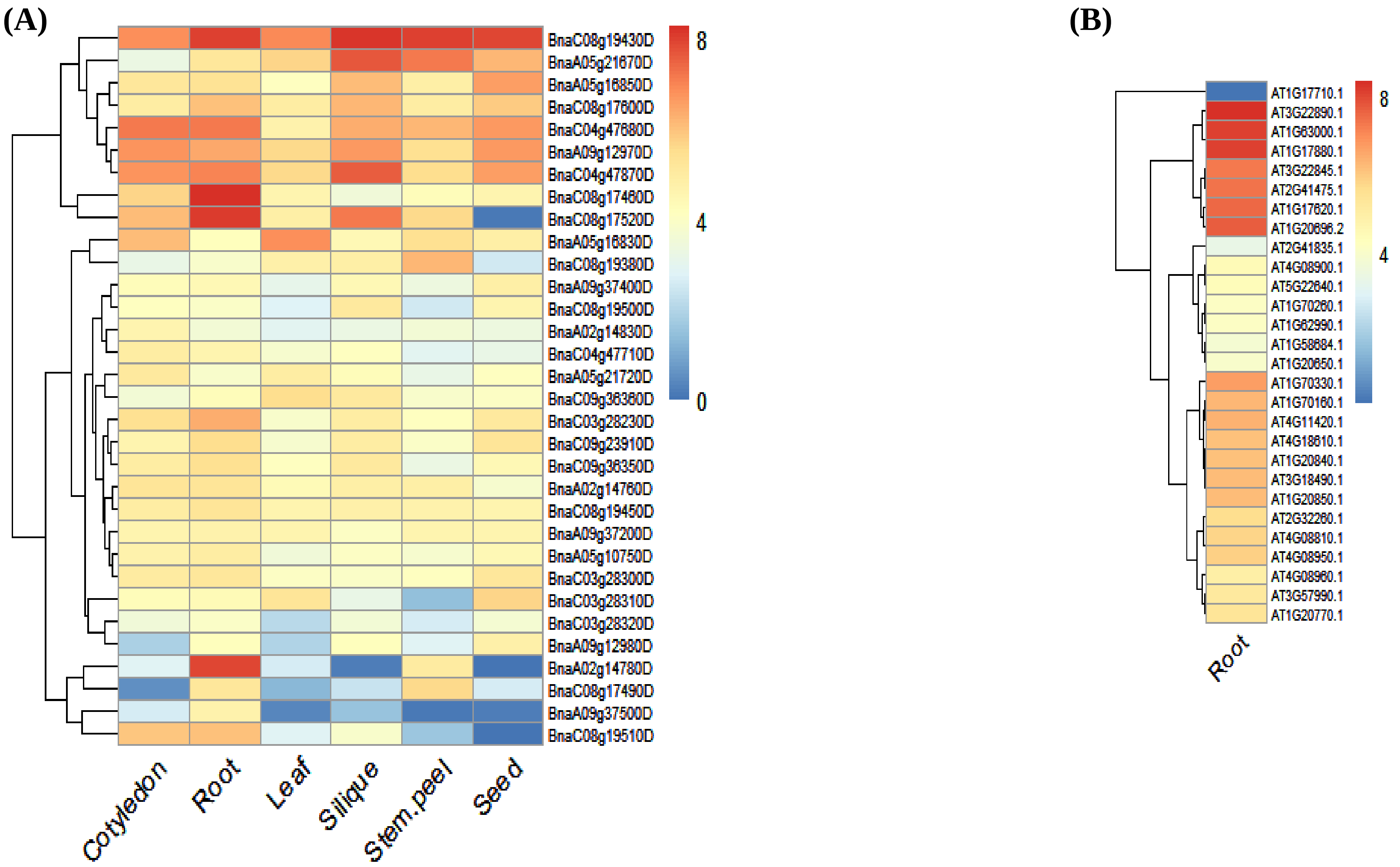

Expression profile of candidate genes. (A) *Brassica napus* expression profiles of potential genes in six distinct tissues. (B) *Arabidopsis* homologous gene expression profiles. The heat map is based on the log2(TPM+1) values. The redder the color, the more linked genes are overexpressed. Genes are grouped via hierarchical clustering

Figure S5

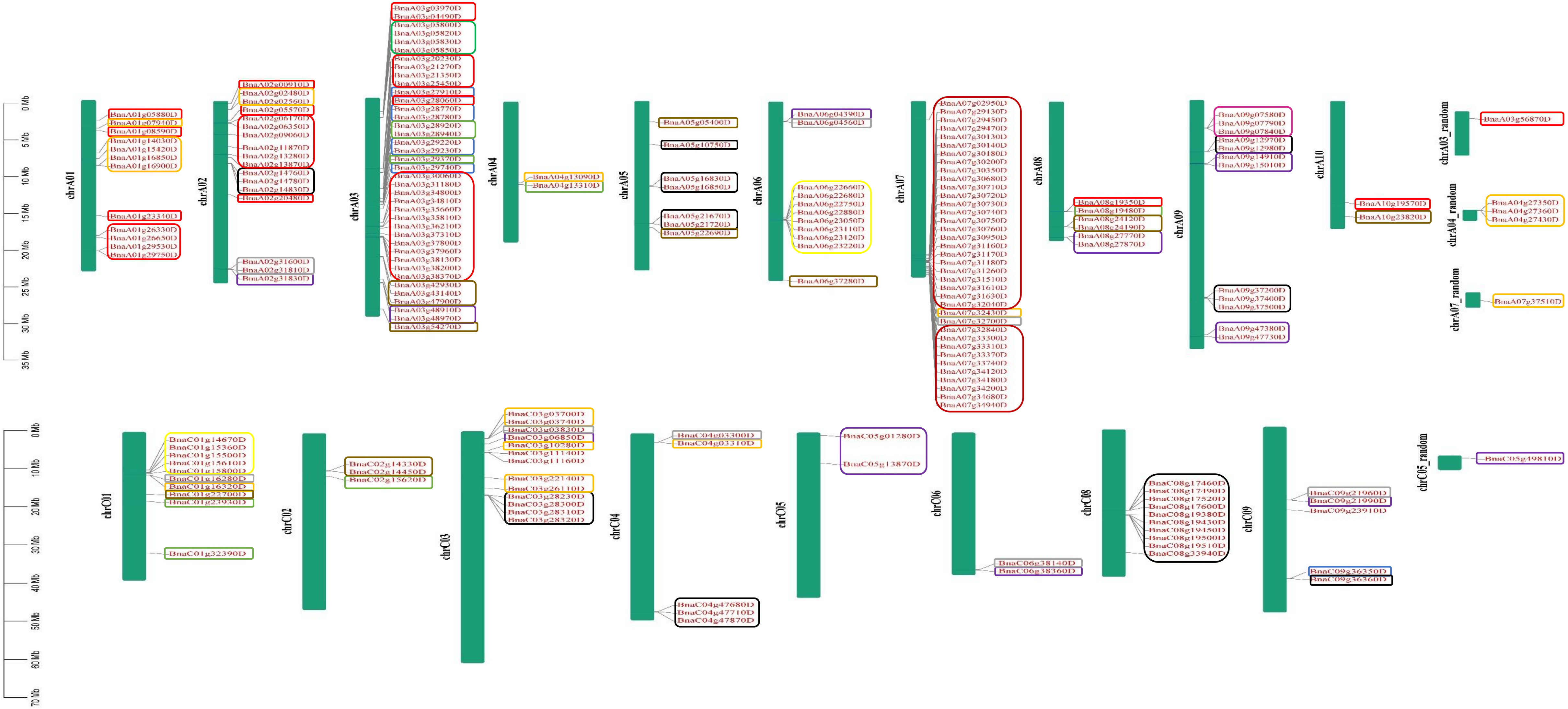

Distribution of our and previously reported candidate genes for root growth and development across 18 of *Brassica napus*' 19 chromosomes. Our candidate genes were indicated by black boxes; Gray, Arifuzzaman *et al.*, 2020b; Blue, Zhange et al., 2015; Green, He *et al.*, 2019; Red, Fletcher *et al.*, 2016; Purple, Arifuzzaman *et al.*, 2019; Orange, Arifuzzaman *et al.*, 2020a; Yellow, Duan *et al.*, 2021, Pink, Wang *et al.*, 2017; Gold, Li *et al.*, 2021; Dark red, Wang *et al.*, 2019
