# Supplementary material for: Genetic Dissection of Mature Root Characteristics by Genome-Wide Association Studies in Rapeseed (Brassica napus L.)
Source: Plants (Basel). 2021 Nov 24;10(12):2569. doi: 10.3390/plants10122569 (PMC8705616; doi:10.3390/plants10122569)
Supplement: Supplementary file 1 [file plants-10-02569-s001.zip › Supplementary file 3.pdf]

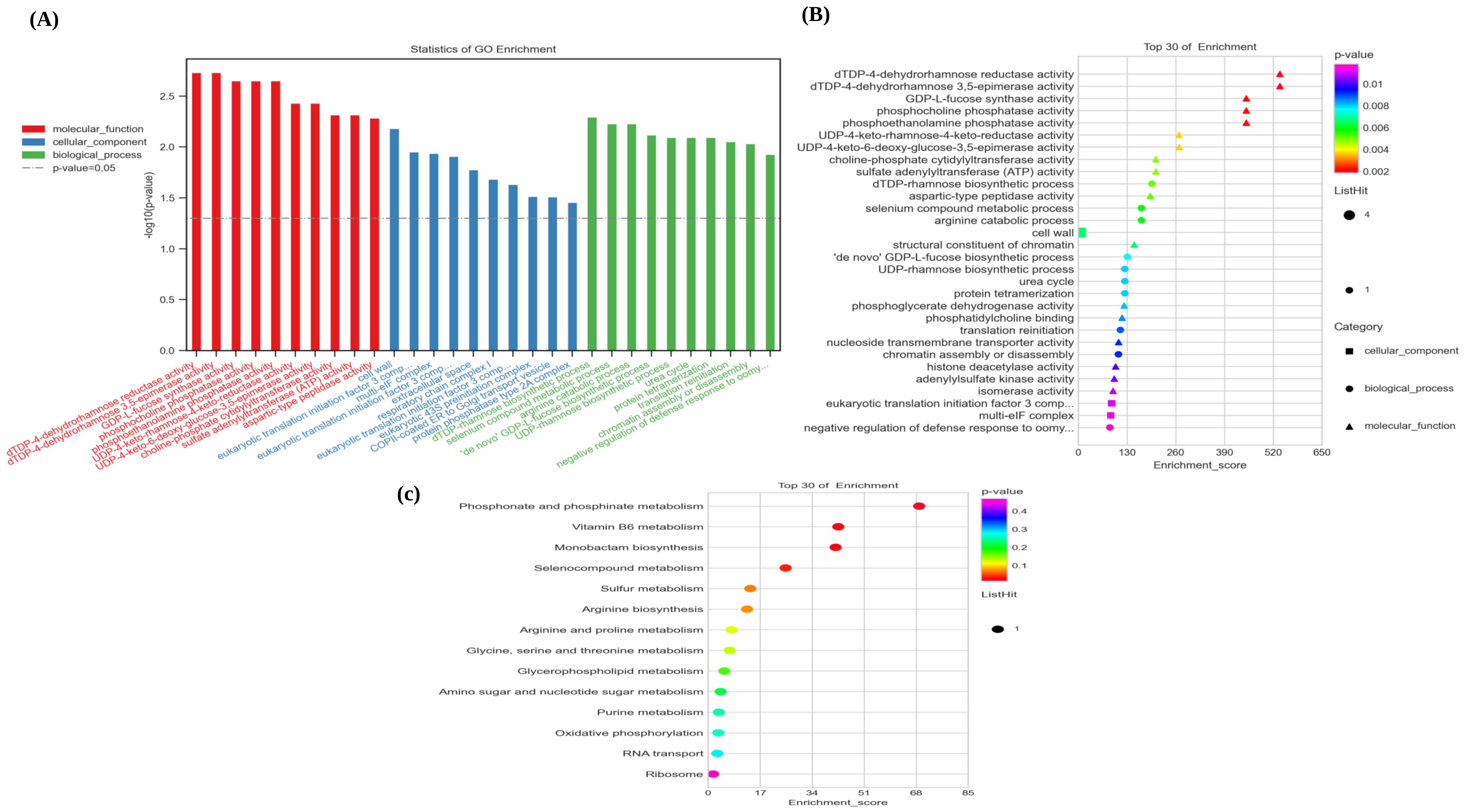

Figure S6. Functional annotation of the detected candidates genes. (A) GO classification of the genes (B) GO analysis of the top 30 genes (C) KEGG analysis of the top 30 genes
