# Supplementary material for: Genetic Dissection of Mature Root Characteristics by Genome-Wide Association Studies in Rapeseed (Brassica napus L.)
Source: Plants (Basel). 2021 Nov 24;10(12):2569. doi: 10.3390/plants10122569 (PMC8705616; doi:10.3390/plants10122569)
Supplement: Supplementary file 1 [file plants-10-02569-s001.zip › Supplementary file 4.pdf]

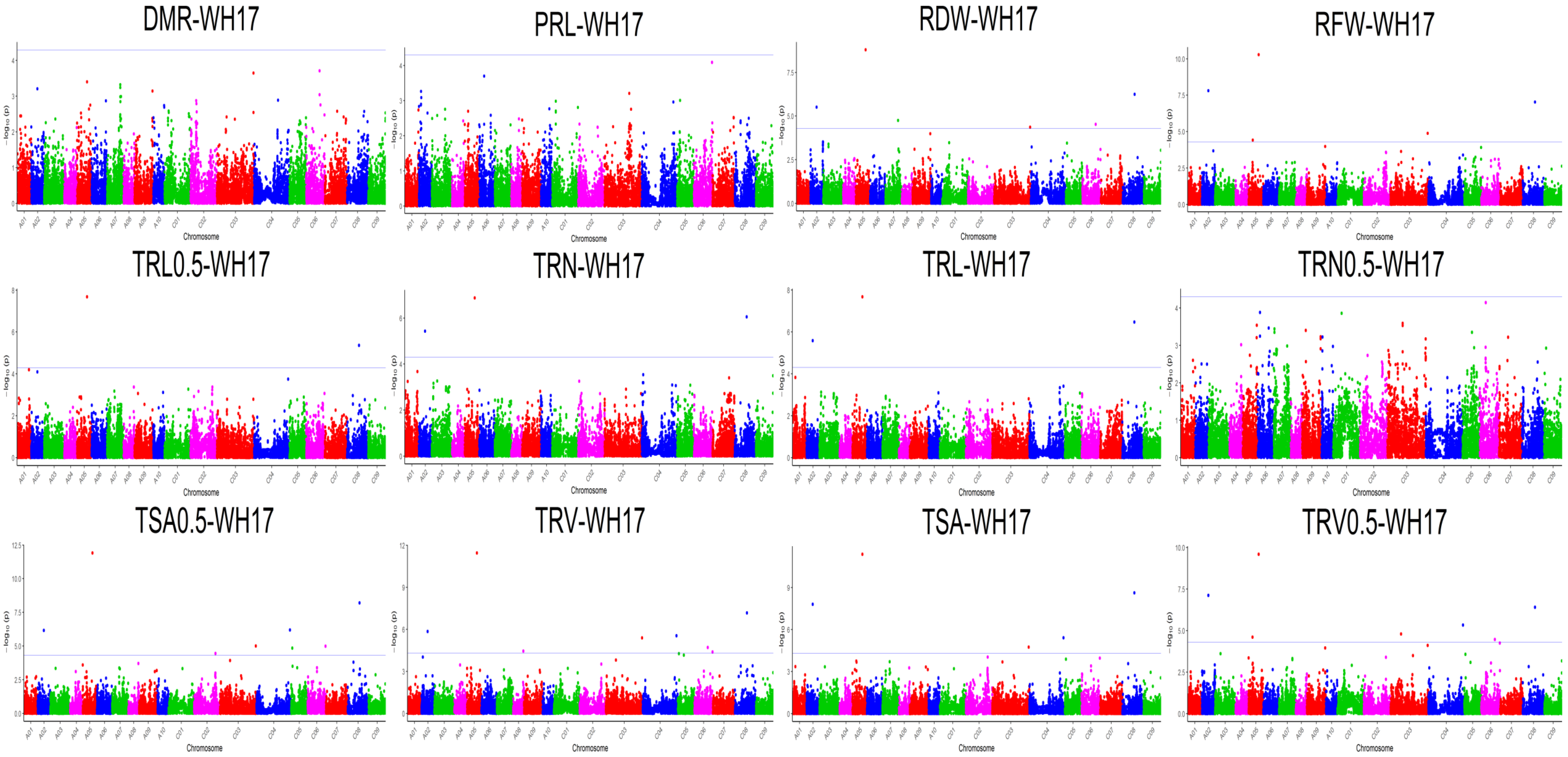

Figure S7A. Manhattan of phenotype-genotype association analysis for twelve root-related traits of *B. napus* by MLM in WH17. The different colors in the plots indicate the 19 chromosomes from A01 to C09; the thick horizontal lines represent the threshold values ($-\log_{10}1/20{,}131$ = 4.30E-5). The colors dots above the threshold values indicate the significant SNPs

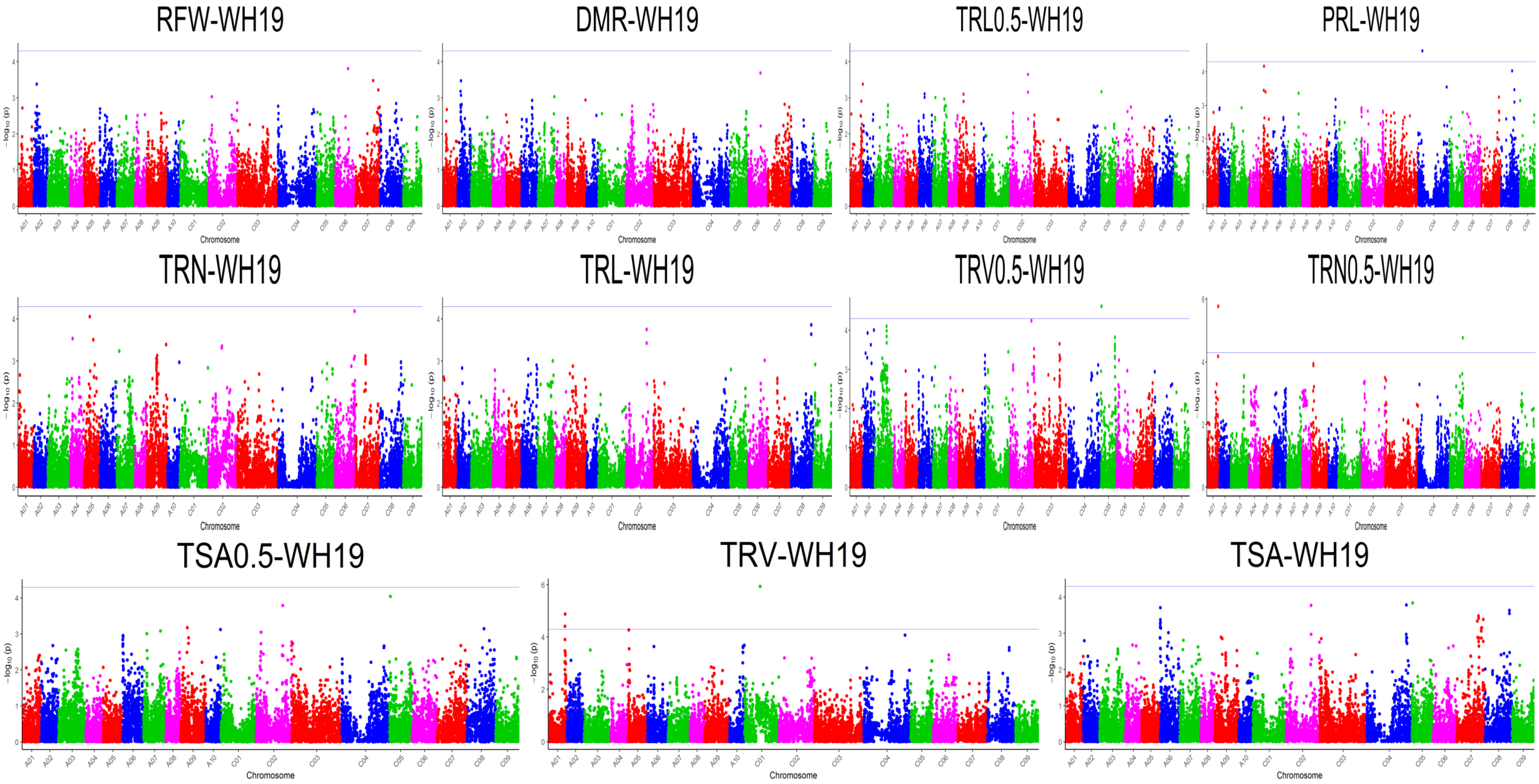

Figure S7B. Manhattan of phenotype-genotype association analysis for twelve root-related traits of *B. napus* by MLM in WH19. The different colors in the plots indicate the 19 chromosomes from A01 to C09; the thick horizontal lines represent the threshold values ($-\log_{10}1/20{,}131$ = 4.30E-5). The colors dots above the threshold values indicate the significant SNPs

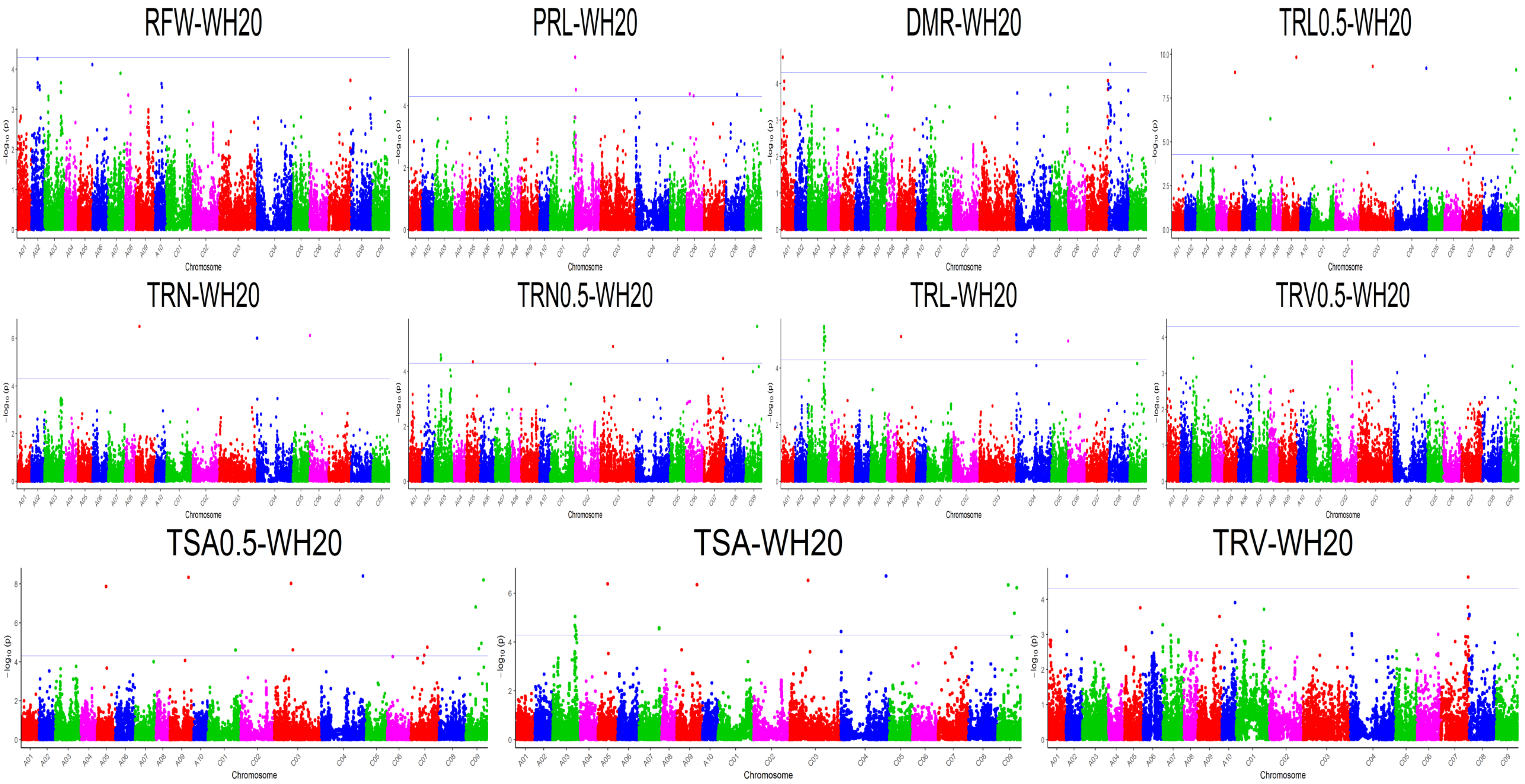

Figure S7C. Manhattan of phenotype-genotype association analysis for twelve root-related traits of *B. napus* by MLM in WH20. The different colors in the plots indicate the 19 chromosomes from A01 to C09; the thick horizontal lines represent the threshold values ($-\log_{10}1/20{,}131 = 4.30E\text{-}5$). The colors dots above the threshold values indicate the significant SNPs

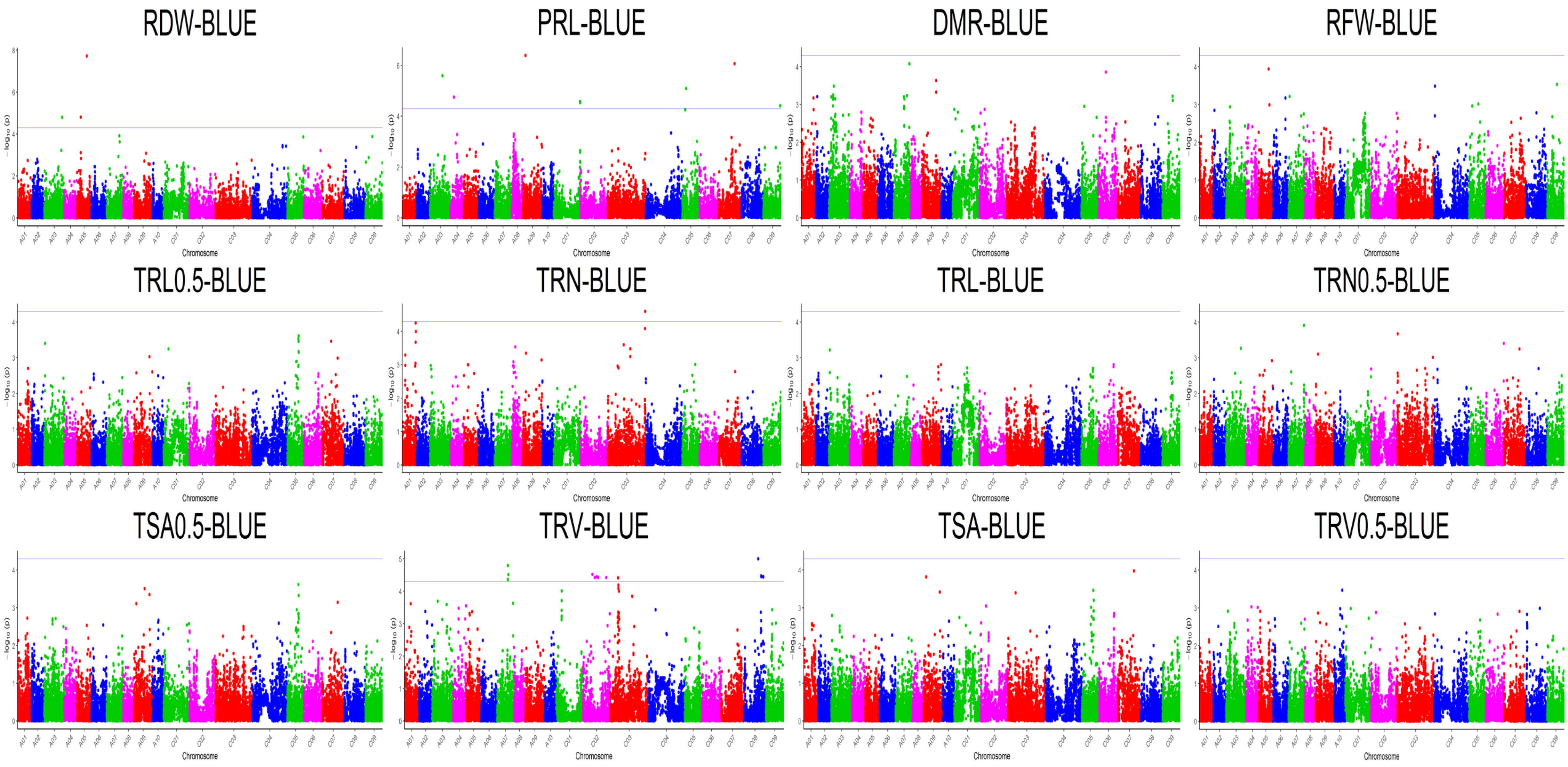

Figure S7D. Manhattan of phenotype-genotype association analysis for twelve root-related traits of *B. napus* by MLM using BLUEs. The different colors in the plots indicate the 19 chromosomes from A01 to C09; the thick horizontal lines represent the threshold values ($-\log_{10}1/20{,}131 = 4.30E-5$). The colors dots above the threshold values indicate the significant SNPs
