# Supplementary material for: Genetic Dissection of Mature Root Characteristics by Genome-Wide Association Studies in Rapeseed (Brassica napus L.)
Source: Plants (Basel). 2021 Nov 24;10(12):2569. doi: 10.3390/plants10122569 (PMC8705616; doi:10.3390/plants10122569)
Supplement: Supplementary file 1 [file plants-10-02569-s001.zip › Supplementary file 5.pdf]

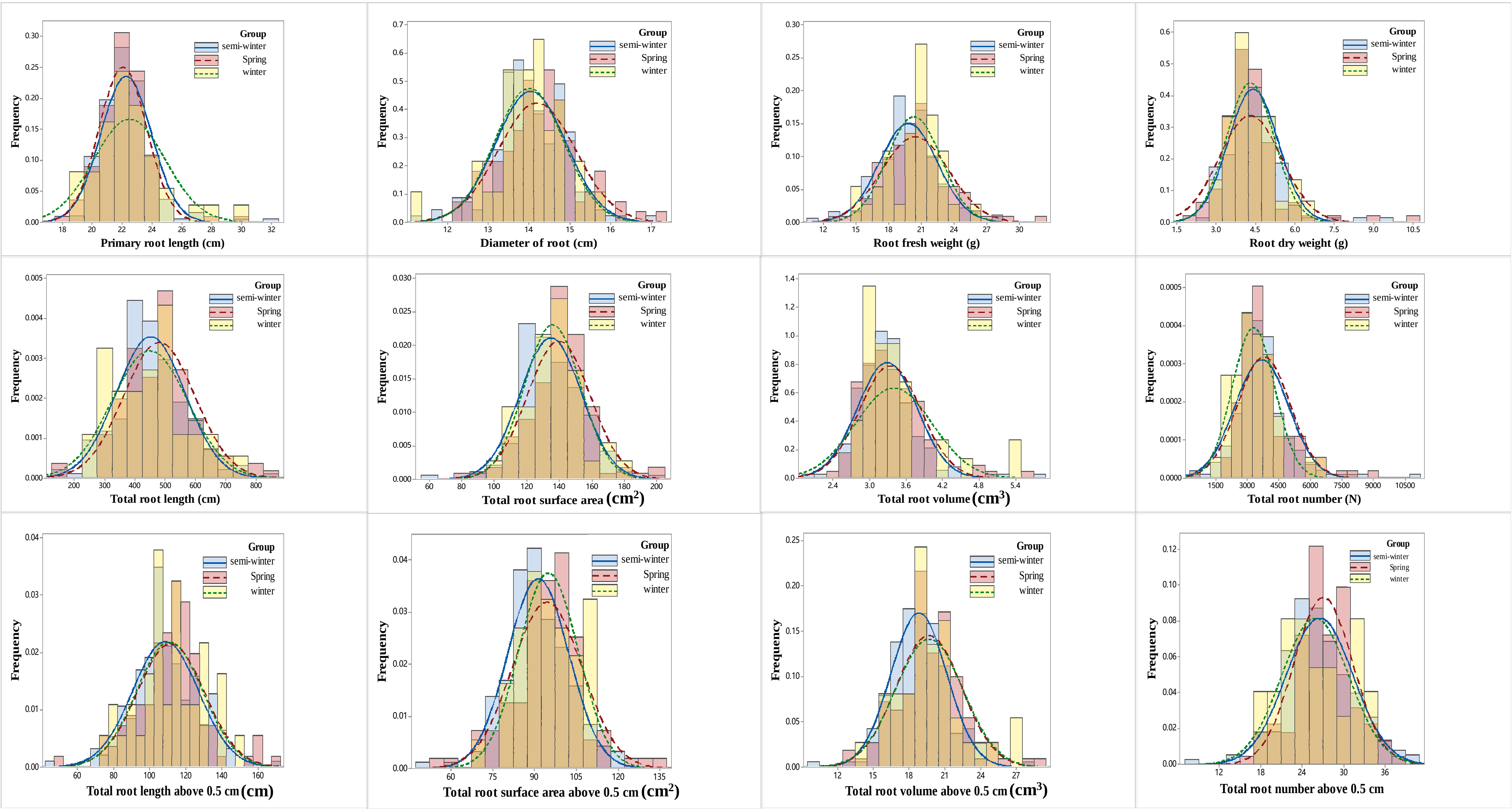

Figure S8. The diversity collection of *Brassica napus* revealed distribution in three subpopulations: winter rapeseed, spring rapeseed, and semi-winter rapeseed. The subpopulations are represented by the three colors of the bars.
